# A dimensionless number for understanding the evolutionary dynamics of antigenically variable RNA viruses

**DOI:** 10.1098/rspb.2011.0435

**Published:** 2011-05-04

**Authors:** Katia Koelle, Oliver Ratmann, David A. Rasmussen, Virginia Pasour, Jonathan Mattingly

**Affiliations:** 1 Department of Biology, Duke University, PO Box 90338, Durham, NC 27708, USA; 2 Fogarty International Center, National Institutes of Health, Bethesda, MD 20892, USA; 3 Department of Infectious Disease Epidemiology, Imperial College London, Norfolk Place, London W2 1PG, UK; 4 Department of Mathematics, Duke University, PO Box 90230, Durham, NC 27708, USA

**Keywords:** phylodynamics, infectious disease dynamics, viral evolution, immune escape, antigenic evolution

## Abstract

Antigenically variable RNA viruses are significant contributors to the burden of infectious disease worldwide. One reason for their ubiquity is their ability to escape herd immunity through rapid antigenic evolution and thereby to reinfect previously infected hosts. However, the ways in which these viruses evolve antigenically are highly diverse. Some have only limited diversity in the long-run, with every emergence of a new antigenic variant coupled with a replacement of the older variant. Other viruses rapidly accumulate antigenic diversity over time. Others still exhibit dynamics that can be considered evolutionary intermediates between these two extremes. Here, we present a theoretical framework that aims to understand these differences in evolutionary patterns by considering a virus's epidemiological dynamics in a given host population. Our framework, based on a dimensionless number, probabilistically anticipates patterns of viral antigenic diversification and thereby quantifies a virus's evolutionary potential. It is therefore similar in spirit to the basic reproduction number, the well-known dimensionless number which quantifies a pathogen's reproductive potential. We further outline how our theoretical framework can be applied to empirical viral systems, using influenza A/H3N2 as a case study. We end with predictions of our framework and work that remains to be done to further integrate viral evolutionary dynamics with disease ecology.

## 1. Introduction

**Figure 1.**
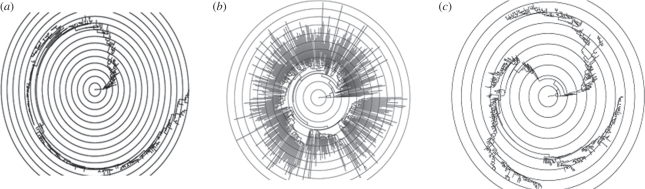
Phylogenetic trees of several antigenically variable RNA viruses. (*a*) Influenza A/H3N2's HA phylogeny in humans, inferred from sequences isolated between 1968 and 2003. (*b*) HIV's gp120 phylogeny (C2-V5 region), inferred from sequences isolated in the US between 1981 and 2007. (*c*) Influenza B's HA phylogeny, inferred from sequences isolated between 1973 and 2008. In all subplots, concentric circles measure time.

Dramatic increases in the amount of viral sequence data have enabled researchers to describe the evolutionary dynamics of specific viral pathogens in ever greater detail over the last several decades. What we are still largely lacking, however, is a synthetic understanding of the diversity of viral evolutionary patterns that are empirically observed [1]. Here, we seek to address this gap by developing a theoretical framework that relates ecological factors to the dynamics of viral antigenic evolution. We specifically focus on antigenically variable RNA viruses, whose surface proteins can exhibit starkly different evolutionary dynamics, as is evidenced by the topological differences in their phylogenies (e.g. figure 1*a*–*c*). An understanding of the causes of these evolutionary differences is necessary to anticipate how these viruses, and the ones that will emerge in the future, will evolve. Moreover, this understanding could lead to novel control strategies that are not simply reactive, but instead aim to shape the evolutionary dynamics of viral pathogens to allow for more effective control.

At the heart of our theoretical framework is a dimensionless number that links herd immunity-driven epidemiological dynamics to an expected rate of antigenic diversification. We base our framework on a dimensionless number because these numbers (e.g. the Reynolds number [2] and the basic reproduction number *R*_0_ [3]) have been shown to be useful in understanding and predicting a system's dynamics, regardless of the specific scale at which the system operates. The dimensionless number *κ* that we propose here for viral evolutionary dynamics is similar to both of these numbers in that its value depends on the properties of two players (the fluid and the pipe in the case of the Reynolds number, and the host and the virus in the case of *R*_0_ and *κ*). It is further similar to *R*_0_ in that its value depends on epidemiological parameters and, ideally, in that it could be used to inform control strategies. The critical difference between *R*_0_ and *κ* is that *R*_0_ quantifies a pathogen's reproductive potential, while *κ* quantifies a virus's antigenic diversification potential.

Our approach is novel for two reasons. First, although classifications of viral phylogenies based on ecological factors such as the degree of immune selection have previously been introduced [4], these have been qualitative in nature, with notable exceptions [5]. Second, previous analyses considering either within-host [6] or population-level [4,5] evolutionary dynamics have classified viral phylogenies into one of the two general types: ‘cactus-like’ (or ‘ladder-like’) phylogenies, with low levels of genetic diversity circulating at any point in time and rapid lineage turnover, and ‘acacia-like’ phylogenies, with growth in genetic diversity over time. Examples of the former include influenza A/H3N2's haemagglutinin (HA) (figure 1*a*) [7,8] and norovirus's capsid protein [9,10], while examples of the latter include HIV's gp120 protein (figure 1*b*) and the HA of influenza A/H5N1 circulating among avian hosts. In contrast to these previous analyses, our *κ* framework considers a spectrum between these diversification patterns. This allows us to quantitatively consider phylogenetic patterns that do not readily fall into one of these two extremes, such as the evolutionary dynamics of influenza B's HA (figure 1*c*).

We organize this paper by first developing our theoretical framework in §2 and then by illustrating the ways in which this framework can be applied to empirical host–virus systems in §3. In §4, we conclude with a discussion of the limitations of our current framework and ways forward towards a better understanding of viral evolutionary dynamics.

## 2. The *κ* framework

In §2*a*, we first describe the structure of the epidemiological model that underlies our theoretical framework and develop our dimensionless number *κ* from this model. In §2*b*, we then show how *κ* can be used to probabilistically describe long-term patterns of antigenic evolution.

### (a) The epidemiological model

We assume that the dynamics of antigenically variable RNA viruses are governed by a status-based multi-strain model [11] of the form:2.1
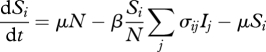
and2.2
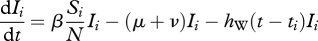
where subscript *i* denotes a single antigenic variant, *μ* is the birth rate and death rate, *β* is the transmission rate, *ν* is the recovery rate and *σ*_*ij*_ is the degree of cross-immunity between antigenic variants *i* and *j*. The term *h*_W_(*t* − *t*_*i*_) represents a *per capita antigenic emergence rate*: the rate at which an individual infected with antigenic variant *i* changes into being infected with a new and yet-unseen antigenic variant [12]. While most evolutionary multi-strain models assume that this rate is constant and equivalent to the mutation rate (or a factor thereof), we here more generally allow *h*_W_ to be a function of an antigenic variant's age *a*, given by the difference between the current time *t* and the time of the variant's emergence *t*_*i*_. Specifically, we parametrize *h*_W_ using a Weibull hazard rate with shape parameter *k*_W_ and scale parameter *λ*_W_:2.3
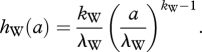


With *k*_W_ = 1, *h*_W_ is a constant. With *k*_W_ > 1, *h*_W_ increases with a variant's age. This can happen if phenotypically neutral mutations accruing over time increase the probability that additional mutations result in significant antigenic change (e.g. [13]) or if a certain number of ‘permissive’ mutations are necessary for phenotypic change to occur (e.g. [14]). The scale parameter *λ*_W_ can be thought of as quantifying the extent of phenotypic robustness to genetic change, with higher values of *λ*_W_ being indicative of higher robustness. As written, equations (2.1)–(2.3) explicitly consider immune selection on viral antigenic phenotypes, rather than on individual genotypes. We use this approach because it yields a computationally simpler model, while remaining consistent with a range of previously published multi-strain model formulations and a range of empirical observations (e.g. [15]). Along with equations (2.1)–(2.3), we assume that a single antigenic variant comprises genotypes that evolve neutrally.

To derive our dimensionless number *κ*, we consider a simplified, stochastic version of this epidemiological model. We start with a single endemic antigenic variant *X* circulating in a host population, giving rise to new variants (‘offspring variants’) over time. We assume that the degree of cross-immunity between variant *X* and an offspring variant is given by *σ*, that there is no cross-immunity between offspring variants, and that offspring variants do not give rise to new antigenic variants. The epidemiological dynamics of variant *X* are governed by classic single-strain susceptible-infected-recovered (SIR) dynamics until an offspring variant emerges that is not stochastically lost from the population. We call this first successful variant *Y*. As a result of variant *Y*'s frequency-dependent selective advantage, and the competition between variants *X* and *Y* for susceptible hosts, the invasion of variant *Y* results in a decrease in the number of individuals infected with variant *X*, until, under a wide range of model parameter values, variant *X* is lost from the host population. Once this happens, the only variants remaining in the population are variant *Y* and any secondary successful offspring variants that may have been generated by variant *X* prior to its exclusion. The dimensionless number *κ* we propose is given by the expected number of these secondary variants (‘excess variants’). Setting the time of emergence of variant *X* to *t*_*X*_ = 0, *κ* is given by:2.4
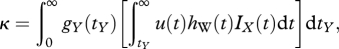
where *I*_*X*_(*t*) is the number of individuals infected with variant *X* at time *t*, *u*(*t*) is the probability that a variant given rise to at time *t* is not stochastically lost from the population and *g*_*Y*_(*t*_*Y*_) is the probability density function for the time to variant *Y*'s emergence.

A conceptually similar quantity was outlined by Sasaki & Haraguchi [6] when considering viral dynamics within chronically infected hosts. Two critical differences, however, exist between the formulation of their dimensionless number and the one we propose here. First, their within-host model assumes that a variant will be driven to increasingly low numbers and ultimately to extinction from a persistent, variant-specific B-cell response. In contrast, an antigenic variant in our population-level model reaches a quasi-stationary endemic steady state when alone in the host population, as long as *R*_0_ > 1. Variant *X* will therefore always generate at least one new successful antigenic variant, variant *Y*, and *κ* quantifies the expected number of *excess* variants that variant *X* generates over its lifetime. Second, Sasaki & Haraguchi's within-host model defines a variant genetically, such that the production of new variants within a host occurs at a constant mutation rate. As described above, our model defines a variant antigenically.

To evaluate *κ* for a given set of epidemiological parameters, we can derive analytical expressions to approximate *I*_*X*_(*t*), *u*(*t*) and *g*_*Y*_(*t*_*Y*_). To derive *I*_*X*_(*t*), we assume that the selective advantage *s* of variant *Y* is constant and given by its initial selective advantage at the time of its emergence *t*_*Y*_. This selective advantage is appropriately defined using variants *Y*'s and *X*'s net reproductive rates. These can be evaluated at time *t*_*Y*_ by analytically solving equations (2.1)–(2.2) for equilibrium when only one variant (variant *X*) is present in the population:2.5
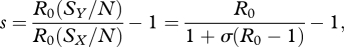
where *S*_*Y*_ and *S*_*X*_ are the number of hosts susceptible to variants *Y* and *X*, respectively, and *R*_0_ = *β*/(*μ* + *ν*). The relative reproductive rate of variant *Y* in units of calendar time is therefore given by *s*(*μ* + *ν*), where 1/(*μ* + *ν*) is an individual's expected duration of infection. Further assuming that the total number of infected individuals (*I*_*X*_ + *I*_*Y*_) stays constant at the equilibrium number of infected individuals for a single-strain SIR system (*I* = *μN*(*R*_0_-1)/*β*), variant *Y*'s population dynamics following its emergence at time *t*_*Y*_ can be written deterministically according to the logistic equation:2.6
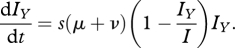


The analytical solution to this equation is:2.7
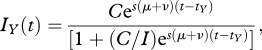
where, with *I*_*Y*_(*t*_*Y*_) = 1, *C* = *I*/(*I* − 1). Our final analytical approximation to *I*_*X*_(*t*) is therefore given by the difference between *I* and *I*_*Y*_(*t*):2.8
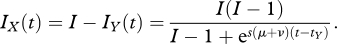


**Figure 2.**
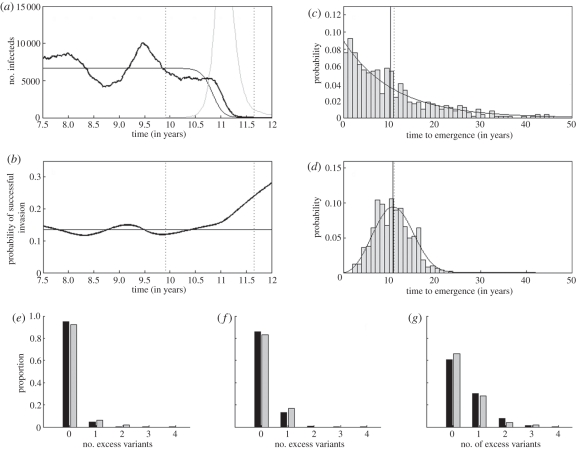
A comparison between the analytical expressions used to calculate *κ* and stochastic simulations of the simplified epidemiological model. (*a*) A representative simulation showing the dynamics of variant *X* (bold black) and variant *Y* (grey), alongside the analytical expression for *I*_*X*_(*t*) (black), given by equation (2.8). (*b*) For the simulation shown in (*a*), the probability that a new variant, emerging at time *t*, successfully invades (*u*(*t*), bold black) alongside its assumed analytical value given by equation (2.9) (black). *u*(*t*) is calculated from the simulation as 1 − 1/(*R*_0_(*S*(*t*)/*N*)), where *S*(*t*) is the number of individuals susceptible, at time *t*, to an offspring variant that has not yet emerged. Simulation parameters are *N* = 18 million, *R*_0_ = 10, *ν* = 1/6 d^−1^, *μ* = 1/40 yr^−1^ and *σ* = 0.85. In both (*a*) and (*b*), the first vertical line shows the time of variant *Y*'s emergence and the second vertical line shows the time of variant *X*'s exclusion. (*c*) The distribution of times to the emergence of variant *Y* from 500 simulations of the simplified epidemiological model (bars), alongside the probability density function, *g*_*Y*_(*t*). Simulation parameters are as in (*a*,*b*), with additional parameters *k*_W_ = 1 and *λ*_W_ = 10 000. Vertical solid black line shows the mean of the 500 simulated times to emergence. Vertical dotted line shows the analytical expected time to emergence (equation (2.13)). (*d*) Same as in (*c*), only with *k*_W_ = 3 and *λ*_W_ = 120. (*e*–*g*) Distributions for the number of excess variants, from 100 simulations of the simplified epidemiological model (grey bars), alongside Poisson distributions with mean *κ* (black bars). In all three subplots, simulation parameters were *N* = 100 million, *R*_0_ = 10, *ν* = 1/10 d^−1^, *μ* = 1/70 yr^−1^, *σ* = 0.9 and *λ*_W_ = 2000. (*e*) Number of excess variants from 100 simulations, each with *k*_W_ = 3.34, yielding *κ* = 0.05. The mean number of excess variants, computed from the simulation results, is 0.09. (*f*) Number of excess variants from 100 simulations, each with *k*_W_ = 2.01, yielding *κ* = 0.15. The mean number of excess variants, computed from the simulation results, is 0.17. (*g*) Number of excess variants from 100 simulations, each with *k*_W_ = 1.476, yielding *κ* = 0.50. The mean number of excess variants, computed from the simulation results, is 0.42. The number of excess variants for a given simulation was calculated by subtracting one from the number of variants co-circulating in the population 5 years after the extinction of variant *X*. We used a likelihood-ratio test to determine whether the null hypothesis (that the number of excess variants comes from a Poisson distribution with mean *κ*) could be rejected at the 95% confidence level in any of the three cases (*e*–*g*) above. In none of the three cases could this null hypothesis be rejected.

In figure 2*a*, the dynamics of variants *X* and *Y* from a representative stochastic simulation are shown alongside the analytical expression for *I*_*X*_(*t*) given by equation (2.8).

To derive *u*(*t*), we rely on the well-known result that the probability of stochastic loss of an invading virus is given by the inverse of its net reproductive rate [16]. Assuming that the number of individuals susceptible to infection at time *t* by an offspring variant that has not yet emerged can be approximated by the number of individuals susceptible to this variant prior to variant *Y*'s emergence; *u* becomes2.9
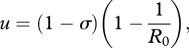
where we have dropped the notation of time dependence. In figure 2*b*, our constant approximation of *u* is shown alongside the dynamics of *u*(*t*) computed from the epidemiological simulation shown in figure 2*a*.

Finally, we derive an analytical approximation to *g*_*Y*_(*t*). This density function can be written as:2.10
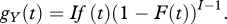
where *f*(*t*) is the probability density function for the time it takes a single individual infected with variant *X* to generate an antigenic variant that is not stochastically lost, and *F*(*t*) is its cumulative distribution function. *f*(*t*) is given by:2.11
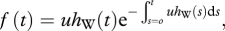
where *u* is the probability given by equation (2.9). Substituting equation (2.3) into equation (2.11) and writing *F*(*t*) in terms of *f*(*t*), equation (2.10) simplifies to:2.12
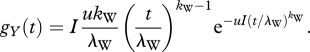


Taking expectations, we have the mean time to the generation of variant *Y*:2.13
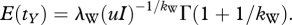
In figure 2*c,d*, we compare our approximation for *g*_*Y*_(*t*) given by equation (2.12) to *g*_*Y*_(*t*) distributions from stochastic simulations of the simplified epidemiological model.

By substituting expressions (2.8), (2.9) and (2.12) into equation (2.4), the value of *κ* can now be computed through numerical integration once all seven epidemiological model parameters (*N*, *μ*, *ν*, *R*_0_, *σ*, *k*_W_ and *λ*_W_) have been specified. As long as *I* and the product *uI* are large, an approximation to this double-integral expression yields:2.14
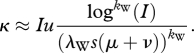


The derivation of this approximation is detailed in the electronic supplemental material. It is clear from equation (2.14) that the epidemiological model parameters affect the final value of *κ* directly and/or indirectly through *I*, *s* and *u*. The sensitivity of *κ* to each of these parameters is illustrated in the electronic supplementary material, figure S1. *κ* increases with population size *N*, with the duration of infection (1/*ν*), with the degree of cross-immunity *σ* and decreases with host lifespan (1/*μ*). Interestingly, the effect of the basic reproduction number *R*_0_ on *κ* depends on the values of the other parameters. Finally, *κ* increases with a decrease in the antigenic emergence rate's scale parameter *λ*_W_ and with a decrease in its shape parameter *k*_W_, most dramatically as *k*_W_ approaches one.

To determine whether *κ* is a good approximation to the mean number of excess variants generated by variant *X* prior to extinction, we simulated the simplified epidemiological model under three different *κ* parametrizations (figure 2*e*–*g*). These simulations show that *κ*, despite the numerous approximations made in its derivation, closely matches the mean number of excess variants computed from simulations. Furthermore, these simulations indicate that the number of excess variants generated by a single endemic variant can be considered a Poisson random variable with mean *κ*.

### (b) The link between *κ* and long-term patterns of viral antigenic evolution

To relate patterns of viral antigenic evolution to *κ*, we first define some useful terminology. Let a generation be defined as the mean time between an endemic variant's emergence time and the emergence time of this variant's first successful antigenic variant offspring. In terms of the simplified model, a generation is given by *E*(*t*_*Y*_). Furthermore, let *n*_*k*_ be defined as the number of antigenic variants co-circulating in a host population in generation *k. *κ** can be used to project the distribution of *n*_*k*_ over successive generations and thereby to describe long-term patterns of viral antigenic evolution under three assumptions: (i) that the time between the emergence of the offspring variant(s) and the extinction of the variant that gave rise to it/them is negligible when compared with the generation time, (ii) that successful antigenic variants immediately equilibrate to their endemic levels, given by *I*, and (iii) that the number of excess variants generated by a single endemic variant is a Poisson random variable with mean *κ*, which we have already seen to be a reasonable assumption (figure 2*e*–*g*).

**Figure 3.**
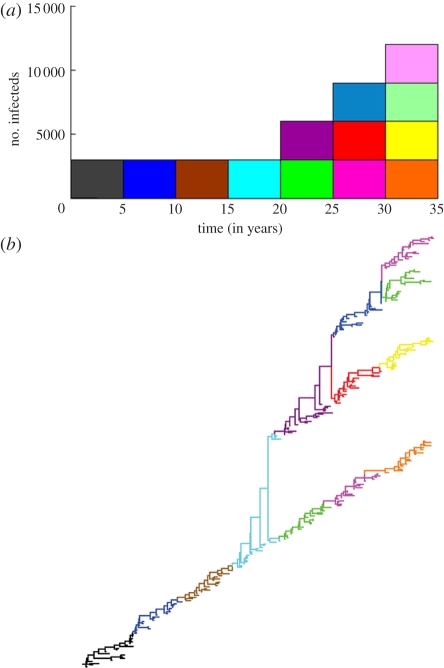
Long-term patterns of antigenic evolution and their dependence on *κ*. (*a*) A single stochastic realization of long-term antigenic evolution, given a *κ* value of 0.2, an equilibrium number of *I* = 3000 infected individuals and a generation time of 5 years. Different colours represent different antigenic variants. Over the first three generations, none of the endemic variants generate any excess variants. In the fourth generation, a single excess variant is generated. In the fifth generation, one of the two endemic variants generates no excess variants while the other generates one excess variant. In the sixth generation, two of the three endemic variants generate no excess variants while the third generates one excess variant. (*b*) A representative phylogeny arising from the diversification dynamics shown in (*a*). The phylogeny was constructed using a neutral coalescent model to generate the branching structure within clusters while the ancestral relationships among clusters and their emergence and death times were determined by the dynamics shown in (*a*). The topology represents one realization of the phylogenetic patterns possible under the given epidemiological dynamics. Colours of the branches correspond to the variant designations shown in (*a*).

Given these assumptions, we have a forward model for simulating antigenic evolution. Starting with only a single endemic antigenic variant (*n*_0_ = 1), *n*_1_ = 1 + *i*_0_, where *i*_0_ is a random variable drawn from a Poisson distribution with mean *κ*. Continuing, the next generation then has *n*_2_ = *n*_1_ + *i*_1_, where *i*_1_ is the sum of a collection of *n*_1_ independent random variables all drawn from a Poisson distribution with mean *κ*. Continuing further, this process results in a synchronized accumulation of antigenic diversity over viral generations. Figure 3*a* illustrates a stochastic realization of these viral diversification dynamics for *κ* = 0.2. Given our model's specification that viral genotypes belonging to a single antigenic phenotype evolve neutrally, we can sample a representative phylogeny arising from these diversification dynamics using coalescent theory (figure 3*b*).

The benefit of *κ* being dimensionless now becomes evident. Although the epidemiological parameters and generation times of two host–virus systems may differ from one another, these systems will be governed by the same diversification rates if they have the same *κ* value. Epidemiologically distinct host–virus systems may therefore exhibit what is known as ‘dynamic similarity’, with similar patterns of antigenic evolution arising from a quantitative agreement of their dimensionless numbers.

From the structure of our forward model, we can also compute the probability mass function for the number of co-circulating variants in a given generation using only *κ*:2.15
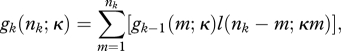
where *l*(*i*;*x*) is the probability that there are exactly *i* variants from a Poisson distribution with mean *x*, *g*_0_(*n*_0_ = 1; *κ*) = 1 and *g*_0_(*n*_0_; *κ*) = 0 for all *n*_0_ ≠ 1.

It is clear from this expression and from the structure of the forward model that a large number of generations may pass in systems with small *κ* before a substantial amount of viral diversity accrues. However, the mean of *n*_*k*_ is given by (1 + *κ*)^*k*^, and its standard deviation grows similarly with the number of generations *k*. The number of co-circulating variants will therefore always end up growing exponentially. According to this model, all viral populations will therefore eventually have many co-circulating variants, and the level of antigenic diversity will not saturate.

The relationship between *κ* and the number of co-circulating antigenic variants in a given generation rests on the three assumptions listed above. To determine our framework's ability to anticipate patterns of antigenic evolution in light of these simplifications, we compared antigenic diversity levels predicted by equation (2.15) against those observed from full simulations of equations (2.1)–(2.3), indicating good qualitative agreement (electronic supplementary material, figure S2). These simulations also show that a range of viral diversification patterns (cactus-like, acacia-like and intermediate phylogenetic tree shapes) can be reproduced under this framework. In the electronic supplementary material, we also further detail how the *κ* framework can be modified for a broader set of strain interactions.

## 3. Applying the *κ* framework to empirical host–virus systems

Here, we detail two ways in which the *κ* framework can be used to understand and predict the evolutionary dynamics of viruses in empirical systems, using influenza A/H3N2 circulating in humans as a case study.

### (a) Estimating *κ* from a phylogeny with antigenic classification of viral isolates

**Figure 4.**
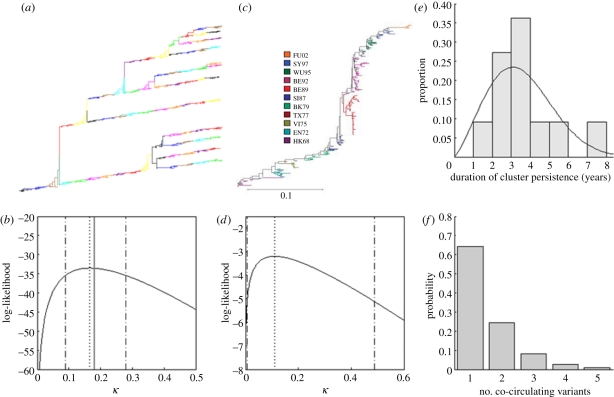
Applying the *κ* framework to the empirical host–virus systems. (*a*) An antigenically-typed phylogeny that was simulated using the stochastic forward model described in §2*b*. The model was simulated for 16 generations using a *κ* value of 0.18. (*b*) Log-likelihood values for a range of *κ* values, calculated from the likelihood expression provided in the text. Solid vertical line indicates the true value of *κ* = 0.18, dotted line is the maximum-likelihood estimate of *κ* and the dashed lines are the 95% CI. (*c*) An antigenically-typed phylogeny of influenza A/H3N2 from sequences isolated between 1968 and 2003. Reproduced from Koelle *et al*. [13]. (*d*) Log-likelihood values for a range of *κ* values calculated from the distribution of excess variants deduced from the phylogeny in (*c*). (*e*) The duration of time each antigenic cluster persisted, defined as the amount of time between when a cluster emerges and when it generates its first successful offspring variant. The mean cluster persistence time is 3.51 years. Black curve shows the *g*_*Y*_(*t*) for the best estimate of *k*_W_ and *λ*_W_, calculated as follows: *I* was first computed from estimates of the annual attack rate, *N* and *ν*, *u* was then computed using equation (2.9), given estimates of *R*_0_ and *σ*. Using these values of *I* and *u*, and for given values of *k*_W_ and *λ*_W_, the probability of observing each of the estimated cluster persistence times can be calculated using equation (2.12). The maximum-likelihood estimates of *k*_W_ and *λ*_W_ were found by searching (*k*_W_ and *λ*_W_) parameter space for the maximum product of these probabilities, yielding *k*_W_ = 2.26 and *λ*_W_ = 1162. (*f*) The probability of observing a given number of co-circulating antigenic variants in 2011. These probabilities were computed using equation (2.15), knowing that there was only one circulating variant in 2003 (FU02) and that a generation lasts approximately 3.5 years, and assuming that *κ* = 0.11.

The forward model of antigenic evolution assumes that the number of excess variants generated by an endemic viral variant prior to its extinction is Poisson-distributed with mean *κ*. We can use this assumption to compute the probability of observing a given number of excess variants arising from a single circulating antigenic variant, and multiply these probabilities to compute the overall likelihood of the dimensionless number *κ* from the distribution of observed excess variants. Figure 4*a*,*b* provides an example, with a phylogeny simulated as in figure 3. The antigenically typed phylogeny shows that 60 out of the 72 endemic variants yielded zero excess variants, while the remaining 12 yielded a single excess variant. The likelihood function is therefore given by:2.15
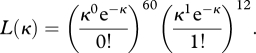
Evaluating this function for *κ* values between 0 and 0.5 clearly shows that our maximum-likelihood estimate of *κ* = 0.167 falls close to the true value of *κ* = 0.18 that was used in the simulation of the phylogeny.

Applying this same approach to influenza A/H3N2 in humans, we see that eight antigenic variants between 1968 and 2003 yielded zero excess variants, and only a single variant, SI87, yielded one excess variant, BE92 (figure 4*c*). (SI87's first successful offspring variant BE89 was lost from the population within a few years.) Our maximum-likelihood estimate of *κ* from this distribution of excess variants is 0.11 (figure 4*d*). Given a mean generation time of approximately 3.5 years (figure 4*e*), we can now use this estimate to probabilistically predict the number of co-circulating antigenic variants in three viral generations, bringing us roughly to today (figure 4*f*). Our framework predicts with a probability of 64 per cent that only a single antigenic variant will be circulating today. This is indeed the case, with antigenic variant Perth/09 currently circulating, having evolved from variant Brisbane/07 [17].

### (b) Estimating *κ* directly from epidemiological parameters

To review, the seven fundamental parameters that are needed to estimate *κ* are *N*, *ν*, *μ*, *R*_0_ (or *β*), *σ* and the antigenic emergence rate's parameters *k*_W_ and *λ*_W_. Host-population size *N*, however, only plays a role in determining the equilibrium number of infected individuals *I*, and host lifespan similarly only plays this role if (*μ* + *ν*) ≈ *ν*. An estimate of *I*, along with estimates of *ν*, *R*_0_, *σ*, *k*_W_ and *λ*_W_, are therefore only required to compute *κ*. Some of these parameters are well-known or routinely estimated. For example, for influenza A/H3N2, 1/*ν* is roughly 3 days, *R*_0_ is roughly 2 [18], *σ* is roughly 0.75 [19,20] and *I* can be estimated by assuming an annual attack rate of 6 per cent and a population size of six billion. This leaves *k*_W_ and *λ*_W_, which can be estimated together from the distribution of cluster persistence times and estimates of *I*, *R*_0_ and *σ* (figure 4*e*). Given these parameter estimates, we compute *κ* = 0.50, at the very top end of the 95% confidence interval (CI) using the method discussed above (figure 4*d*). Clearly, this value of *κ* is an overestimate, and we return to this disparity below.

## 4. Discussion

Here, we proposed a dimensionless number *κ* for understanding and predicting the evolutionary dynamics of antigenically variable viruses. We derived this number from our knowledge of multi-strain epidemiological dynamics and the frequency-dependent selection pressures that arise from these dynamics. The dynamics depend only on a handful of parameters, many of which are known or can be estimated in a straightforward way from empirical data.

The *κ* framework presented here makes several predictions. One set of predictions is provided by the expression of *κ*, given by equation (2.4), approximated by equation (2.14), and depicted graphically in electronic supplementary material, figure S1: with all else equal, we expect a virus to exhibit more rapid antigenic diversification when circulating in a larger host population, when circulating in a shorter-lived host population and when resulting in longer durations of infection. We also expect viruses with lower cross-immunity between antigenic variants to have lower rates of diversification. Finally, we expect the rate of antigenic diversification to strongly depend on the antigenic emergence rate's *k*_W_ parameter, controlling how the rate of antigenic emergence depends on a variant's age. As the effect of ‘ageing’ diminishes, *k*_W_ approaches one, yielding higher *κ* values. This relationship can therefore explain why epidemiological models that do not include this effect (such that *k*_W_ = 1) result in explosive genetic and antigenic diversity in the absence of other factors such as generalized immunity [21], whereas models that include this ageing effect can reproduce cactus-like evolutionary dynamics without invoking other factors [12,13,22].

In our application to influenza, we outlined two approaches that can be used to estimate *κ*. The first was based on an antigenically typed phylogeny and yielded reasonable *κ* estimates. The second was based on independent parameter estimates; in this case our calculation of *κ* was a considerable overestimate. This could have been owing to several reasons. First, our framework assumed a homogeneous, well-mixed host population and thereby ignored geographic subdivision and networks of interaction, which have clearly been shown to play a role in shaping viral phylogenies [23–25] and impacting antigenic diversity [26]. Second, we assumed no seasonality or other factors that could lead to significant variation in the number of infected individuals over time. These factors are likely to reduce the degree of antigenic diversity by increasing the probability of stochastic loss. Finally, viral strains may differ phenotypically in ways unrelated to their antigenicity, and these differences may also have impacted influenza A/H3N2's HA phylogeny. Future work should therefore focus on extending this minimal *κ* framework to improve our ability to predict patterns of antigenic diversification from independent parameter estimates.

Despite these limitations, the *κ* framework presented here was shown to reasonably anticipate the viral evolutionary dynamics of influenza A/H3N2's HA when *κ* was estimated from a phylogeny. Our estimate of *κ*, however, assumed a status-based multi-strain model, which has recently been criticized for its biological implausibility. Although this may seem like a major limitation, alternative multi-strain models (e.g. [27]) can easily be accommodated by modifying subcomponents of *κ* (electronic supplementary material).

From the framework we presented here, we can start to understand why viral phylogenies from certain host–virus systems differ topologically from those in other systems, or why they may have remarkable evolutionary similarities. Through this improvement in our understanding, we hope that *κ* may result in more effective disease prediction and control. For endemic viruses, the dependence of *κ* on modifiable epidemiological parameters already points us towards control strategies that may drive a given virus into an evolutionary regime that can be more effectively controlled. For example, the lengthening of livestock lifespans is expected to significantly lower the rates of antigenic diversification. In conjunction with vaccines that would be more effective and long-lasting, this could therefore lead to dramatic reductions in infection levels. For emerging viral zoonoses, *κ* could be estimated by combining what is known about a naive host population (e.g. its population size) with what is known about the virus from its circulation in a reservoir population (e.g. its duration of infection). This could lead to predictions of how a virus will evolve in a host population prior to its establishment and help us focus our efforts on the viruses that would be difficult to control antigenically if establishment occurred. In sum, the minimal *κ* framework presented here, open to many extensions and modifications, could prove increasingly useful for understanding and controlling the evolutionary dynamics of antigenically variable RNA viruses.
